# COVID-19 and the elaboration of personal plans in + 50: a Brazilian experience

**DOI:** 10.1186/s12889-023-15006-1

**Published:** 2023-02-01

**Authors:** Kerolyn Ramos Garcia, Andrea Pecce Bento, Aline Gomes de Oliveira, Rafaela Alves da Silva, Marileusa Dosolina Chiarello, Isabelle Patriciá Freitas Soares Chariglione, Margô Gomes de Oliveira Karnikowski

**Affiliations:** 1 Postgraduate Programme in Health Sciences and Technologies (PGCTS), University of Brasilia, Faculty of Ceilândia (FCE) Campus Universitário - Centro Metropolitano, Ceilândia Sul, CEP:, 72220-275 Brasília - DF, Brasil; 2 University of Envelhecer (UniSER) of University of Brasilia, Brasilia, Brazil; 3 Support Centre for Technological Development (CDT) of University of Brasilia, Brasilia, Brazil; 4 Post-Graduate Program in Developmental and School Psychology, University of Brasilia, Brasilia, Brazil

**Keywords:** Old adults, Longevity, Future planning, Personal plans

## Abstract

**Background:**

In front of the physical and social isolation, as well as feelings of fear and instability imposed by the pandemic of COVID-19, especially for risk groups such as people 50 + , it became even more relevant to discuss the formulation of personal plans for this age population. This study aimed to analyse the individual plans of people 50 + , considering their perception, feelings and life experiences during the COVID-19 pandemic.

**Methods:**

This is a mixed study (quali-quantitative), using Minayo’s methodology for content analysis and frequency analyses, made with 39 participants from the University of Brasília Educational Program, Universidade do Envelhecer – UniSER/UnB. We used a word cloud system and a wheel of life tool to showcase the results.

**Results:**

Analysing professional achievements and situations participants want to overcome, the categories of feelings that stand out were Loving Relationships, Sadness, Family Absence, Grief, Trauma and Regret. Intellectual Development, Personal Fulfilment, Purpose and Creativity, Hobbies and Fun were the most mentioned personal plans displayed by the wheel of life. The key personal changes mentioned were to be less shy, prioritise themselves, change how they interact with others, and focus on their health.

**Conclusions:**

This study concludes that perception, feelings and life experiences during the COVID-19 pandemic did not seem to directly affect the path in elaborating personal plans.

**Supplementary Information:**

The online version contains supplementary material available at 10.1186/s12889-023-15006-1.

## Background

The privilege of living longer was conquered by humanity and it is associated with improvements in quality of life, physical activity, health technologies, changes in lifestyle, maintenance of dignity and preservation of older peoples’ rights, among others [1–3]. Additional years are also accompanied by the challenges that longevity brings in the cognitive, physical and social fields [4].

As a country with continental dimensions and significant regional differences, Brazil’s population has reached longevity faster than European countries [5, 6]. However, longevity in the country usually comes with negligence of critical aspects of becoming old, with a crescent number of suicides and loneliness, depression, violence and other vulnerabilities constantly used to describe ageing in the country [7, 8]. Besides that, fear of death, illness and loneliness have become more evident in the last years and have been intensified by the COVID-19 pandemic [9], experienced in 2020, still with no conclusions about its durability and with a context of social inequality and social isolation [10, 11]. As the pandemic continues, the scientific focus is now also considering the longer-term fallout from the pandemic [12].

In addition to living longer, it is necessary to live well. As pointed out by several authors, planning old age in its various dimensions is a vital strategy to positively increase the quality of life indicators and promote active and healthy ageing [13, 14]. Making plans in adult and old age is relevant when we consider that, at this phase of life, time is even more precious, especially when this life stage is lived during a pandemic scenario that killed a significant number of 50 + population [15]. The period of social isolation can be used as a moment for reflecting on actions and gives new meaning to human relationships and perspectives on the present and the future. Therefore, COVID-19 made it necessary to stimulate qualitative research about the lived experience of 50 + that are currently passing through the pandemic [16]. It becomes a good time for the 50 + group to think and make reflections on life, consequently planning, being relevant to understand those plans to stimulate the formulation of projects and interventions that address their needs.

Establishing priorities, which will support the planning of actions to be taken in the future, become more relevant, as well as the comprehension of what is more suitable for the 50 + public to be prioritised. In this scenario, the actual generation of 50 + , being aware of their potential, vulnerabilities, and limitations, can expand the dimension of their social, family and professional actions. Nowadays, it is necessary for those who are 50 + to redesign the cycles of their lives, directing their efforts not only to the maintenance of body health but towards the fulfilment of life dreams and social engagement [17, 18], all of those essential aspects to be considered to make personal plans at 50 + lives.

Another critical aspect to consider is life experiences and perceptions about the self and the world. Studies show that feelings and experiences nurtured during the existence of a human being often guide their personal plans [19–21].

## Methods

This paper aims to analyse the personal plans of people 50 + , considering their perceptions, feelings and life experiences during the COVID-19 pandemic.

This work is a mixed qualitative and quantitative research, featuring a content analysis using Minayo’s as reference (qualitative) [22] and considering the frequency analysis for the data that was quantified (quantitative). The survey was carried out within the University of Brasília Program “Aging University—Universidade do Envelhecer” (UniSER/UnB), an educational program headquartered in Brazil’s capital focused on people 50 years old or more, with no schooling restrictions. The initiative focuses on promoting integrative actions guided by the principles of health, education, entitlement, advocacy, art and culture [23]. With the pandemic of COVID-19, the activities of the UniSER/UnB were changed to remote/distance mode.

**Fig. 1:**
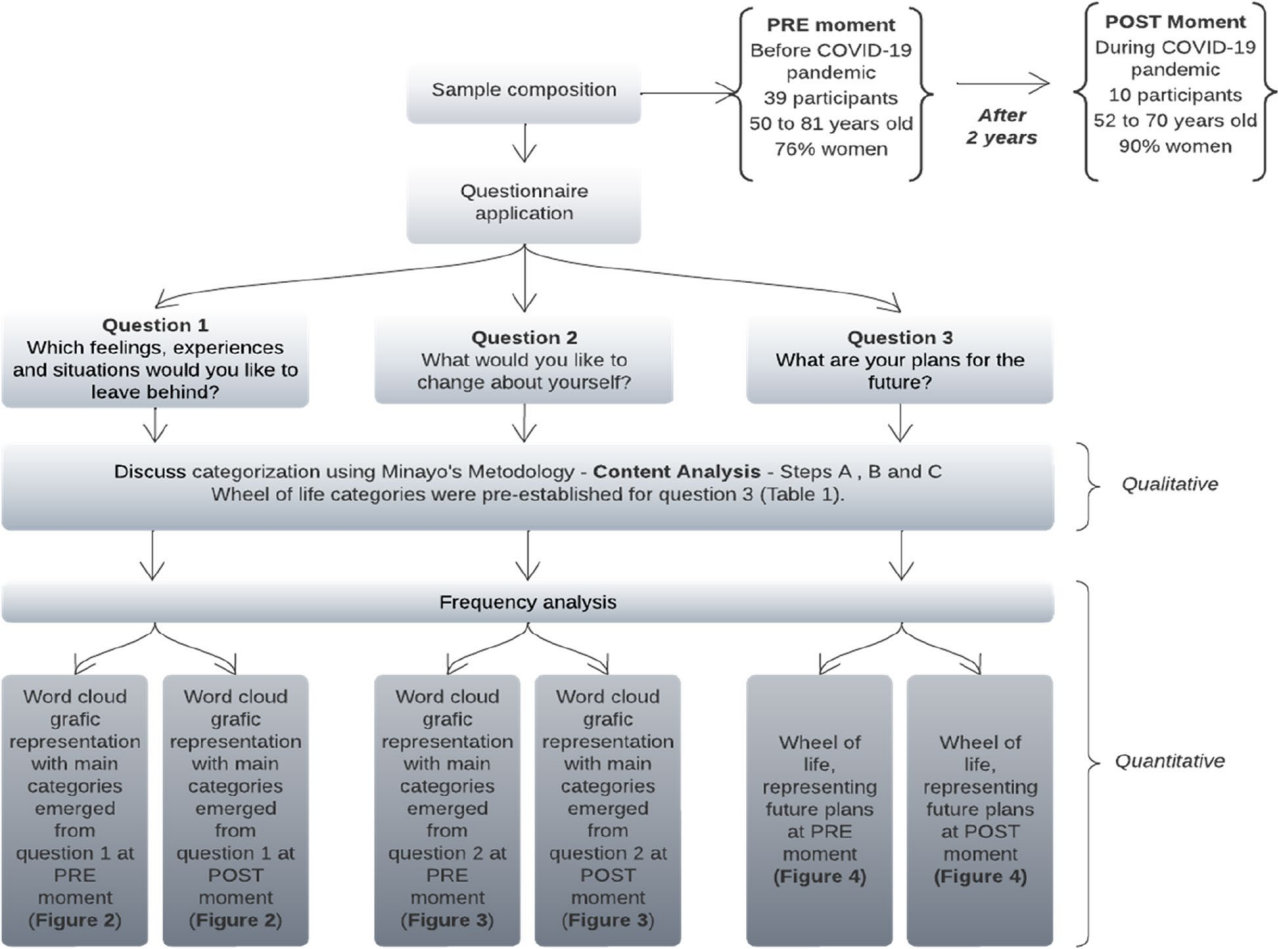
Research steps. Fonte: Own authorship, Brazil, 2022

The survey’s sample was defined by convenience (it was made an open call to the UniSER participants who were interested to participate) and included 39 participants of the UniSER/UnB, with 50 to 81 years old (50–59 years = 14; 60–69 years = 20; 70 +  = 5). After 2 years of the first data survey and during the second year of the COVID-19 pandemic (2021), 10 of the 39 participants answered this study’s questions again (52–59 years = 1; 60–69 years = 7; 70 +  = 2). 90% of the sample that did not participate in the post-COVID phase declined to participate in the post-phase of this study for reasons related to the pandemic, and the other 10% had declared personal reasons. The research steps are presented in Fig. 1.

A questionnaire composed of three questions was distributed to the participants, the questions being the ones shown in Fig. 1. The Portuguese version of the questions is available as Supplementary file. The participants who were involved in our sample were invited to answer these questions on a piece of paper on their first day participating in the UniSER/UnB program at the pre COVID-19 moment, and, two years after that (2021), the research team called them to complete the post-COVID-19 moment, also using a piece of paper to answer the same questions, later sending the paper to the research team.

Steps of content analysis proposed by Minayo [22]: (A) Ordering of data to map the material obtained, re-reading the material and organising the reports; (B) Classification of the data with exhaustive and repeated reading of the texts by a research team, for the next constitution of a corpus of communication, followed by the transversal reading of each body as a cut-out of the registration unit and, finally, the cutting of the most relevant data; (C) Final analysis and elaboration of the analytical categories, phase in which the research objectives and the themes that emerge from the observations are taken into account and the data is articulated with the theoretical framework, defining the analytical categories. All the qualitative analyses were made by six researchers across four meetings to discuss the data, which was analysed by each of them, until there was consensus among researchers about the results. Data, where no agreement was reached, is excluded from the analyses. The analytical categories are the results of the content analysis. It is essential to highlight that multiple people categorising the responses is a strength in this study once the findings’ trustworthiness depends upon being able to describe the analytical process clearly and how many people “*can support each other’s reflexivity, challenge each other’s assumptions and co-construct a description or interpretation of the data”* [24].

**Fig. 2:**
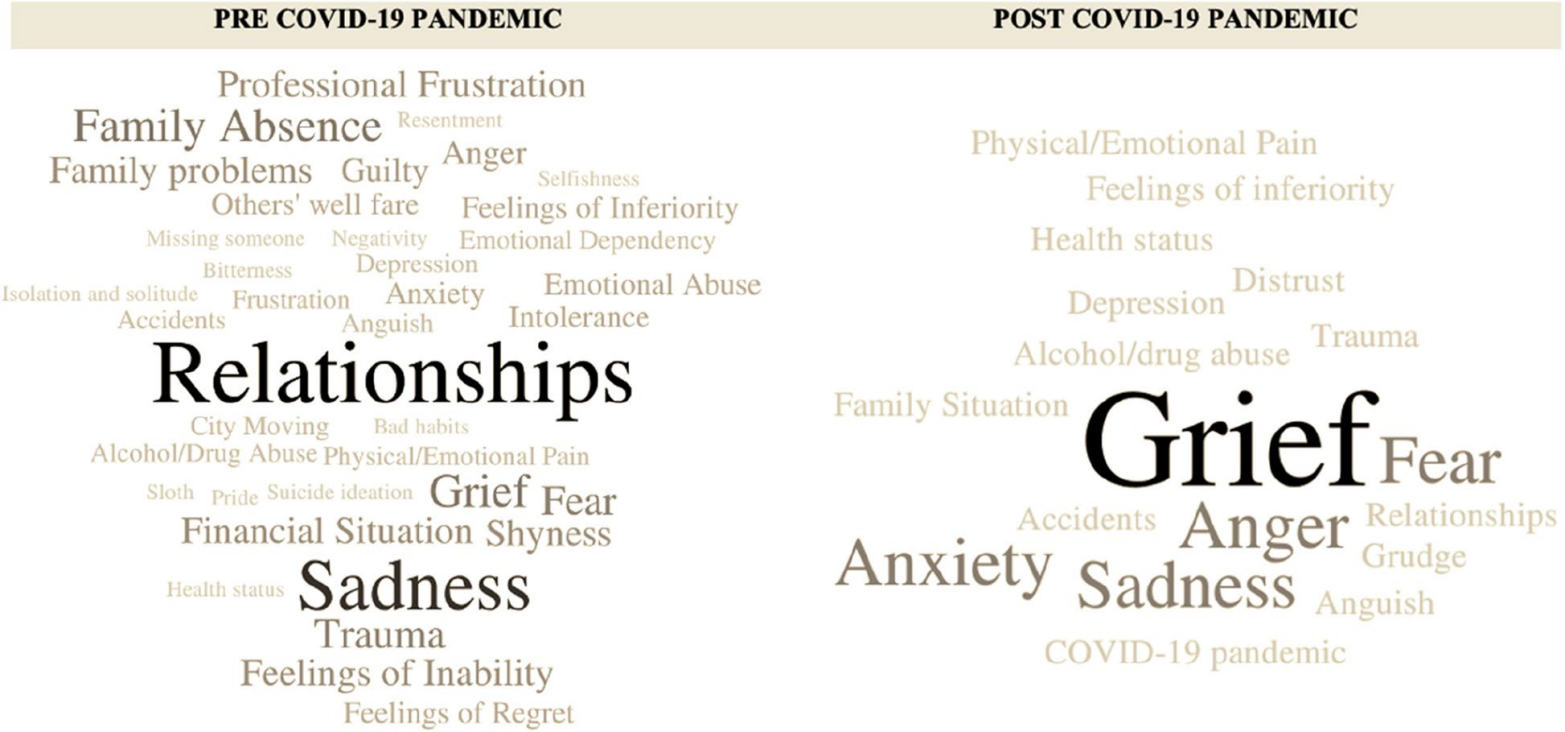
Resulting categories from the sample’s answers about feelings, experiences and situations to be left behind, pre- and post-COVID-19 pandemic. Fonte: Own authorship, word cloud generated by Word it out software, Brazil, 2022

**Fig. 3:**
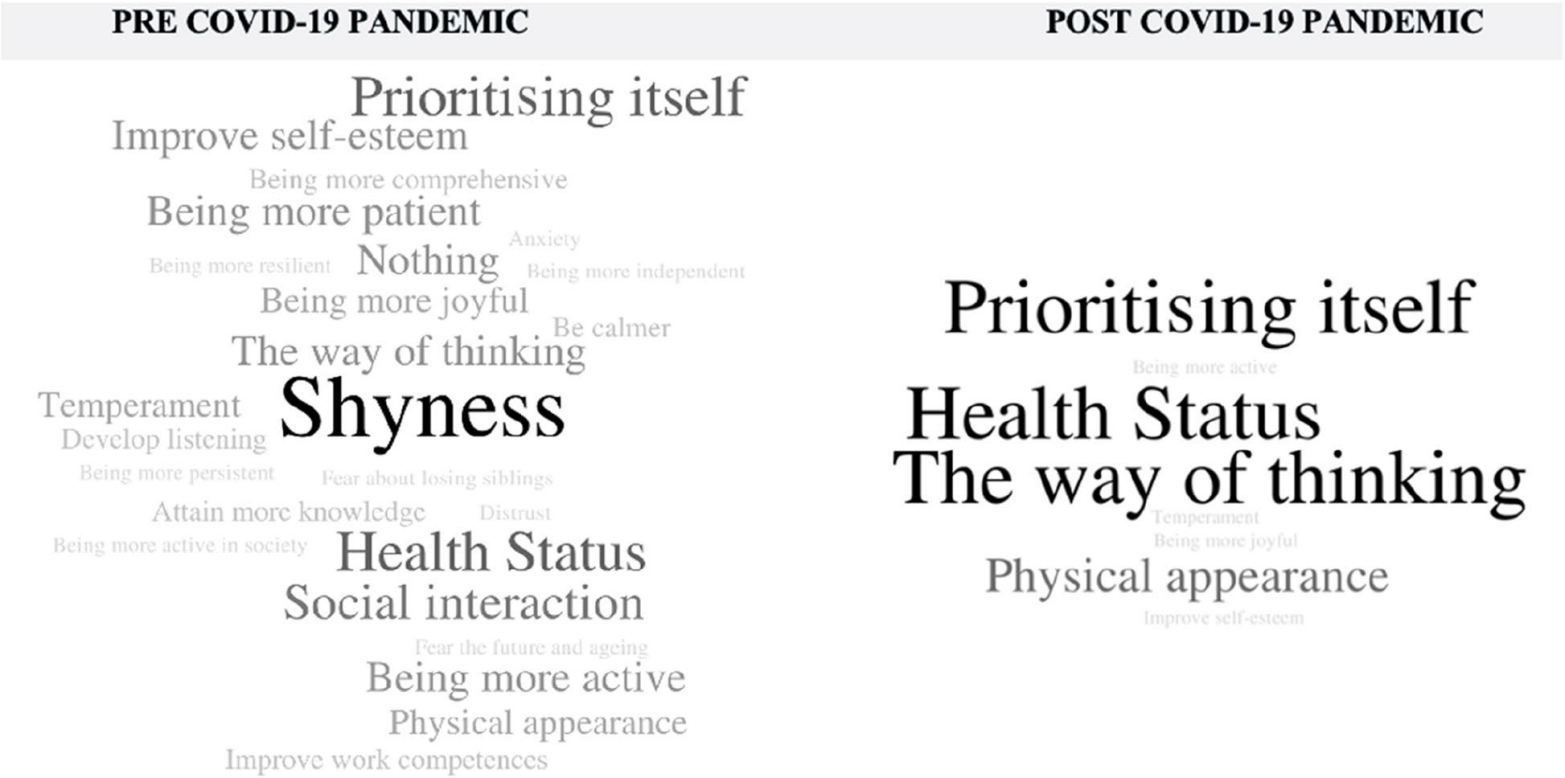
Personal aspects participants would like to change, pre and post-COVID-19 pandemic. Fonte: Own authorship, word cloud generated by Word it out software, Brazil, 2022

The answers to the first and second questions were pooled in pre- and post-moments categories. The main ones were showcased using the *word cloud* graphic representation (Figs. 2 and 3). The word cloud presentation method is a tool that consists of a form of graphic visualisation based on the frequency of words from the participants’ answers [25], which were categorised and released using the free *Word it out* software. The categories have a grey scale of colours and sizes relative to their repetitions. Connectives and words that had no meaning to the research were excluded, as well as the answers that declared no feelings or situations to be left behind. The categories were included in the *word cloud*, using the open software *Word it out* version 2022*.* Both analyses of the two questions included an “others” category, which contemplates all the *feelings, experiences and situations* (question 1) and *characteristics and behaviours* (question 2), referenced only one time.

The *wheel of life* tool, outlined by Torresan [26], was used to pre-determine the categories in which the answers to the third question of the survey were sorted, representing the personal plans resulting from the participants’ answers analysis. The wheel is a coaching instrument used to measure the person’s satisfaction rate in fields considered important to life [27]. Our intention in using this tool was to verify in which categories participants’ plans focused as a group and analyse its results, by sorting them using the *wheel of life’*s classification*.*

The quantitative analyses mentioned in this study refer to the absolute and relative frequency of the words mentioned in the participants' speeches, which gave rise to the construction of the categories noted, as well to construct the presentation of the results of the word cloud and the wheel of life.

The wheel is shaped in a “Mandala” structure and is composed of 4 basic groups: Personal sphere, Professional sphere, Life Quality and Relationships [27]. Within each thematic, there are three indicators, called “triads”, which represent main aspects of each field, making 12 categories total. Because of the wheel’s wide coverage of themes, all the personal plans present in the answers fit in the 12 categories, with one exception of the answer that did not mention personal plans, the answer “*I think that everything that I’ve done was wonderful. Well lived years”*. The categories are showcased and explained in Table 1.

**Table 1:** Definitions adopted in the wheel of life construction, are based on the answers about the participants’ personal plans

Main Aspect	Triad	Definition
***Personal Sphere***	*Intellectual Development*	Investment in Intellectual growth, learning, experiences, studies and increasing knowledge. This included learning not only for professional development but also for growth in maturity
	*Health and Willingness*	Personal care related to mental and physical health and gains of energy, mood, motivation, and vitality
	*Emotional Balance*	Plans related to changing the ways to deal with challenges, conflicts, insecurities, adverse situations, emotional management, and decision-making processes
***Professional Sphere***	*Financial Resources*	Improving financial resources to provide basic living conditions, payment of bills and acquisition of certain products and services. These are the factors related to financial satisfaction as a whole
	*Social Service*	Actions that help change Society such as community and social works and voluntary jobs
	*Purpose and Fulfilment*	Plans related to achieving professional realization and the true reason of being the personal mission
***Life Quality***	*Creativity, Hobbies and Fun*	Planning towards moments of recreation, relaxation, fun and rest for the body and mind as well as activities to recharge batteries, touch their own creativity and to eliminate negative charges
	*Happiness and Feeling of Fullness*	Plans related to the feelings of joy, happiness, enjoyment and experiencing positive moments
	*Spirituality*	Related to spiritual growth and religious beliefs and activities
***Relationships***	*Social Life*	Is related to the ability to connect with different kinds of people and to interact friendly with others
	*Loving Relationship*	Concerning relationships with significant others, emotions that identify your true level of loving commitment and building positive relationships
	*Family*	Relationships with and aspects related to members of the family

Source: Own Authorship

All the answers were originally given in the Brazilian Portuguese language. As a result, some grammar errors such as concordance, punctuation and accentuation mistakes were neither possible nor convenient to translate. The data in Brazilian Portuguese version is available as supplementary file. The data in this study is part of research approved by a Research Ethics Commission, according to the Brazilian National Health Council resolution number 466/12, under procedure number 74646317.8.0000.5056.

## Results

Analyzing the feelings, experiences and situations the participants would like to leave behind pre and post COVID-19 pandemic (Fig. 2), 39 categories stood out for Pre COVID-19 moment: *Relationships* (*n* = 17 citations), *Sadness* (*n* = 13), *Family Absence* (*n* = 7), *Grief* (*n* = 7), *Trauma* (*n* = 6), *Fear* (*n* = 6), *Professional Frustration* (*n* = 5), *Feelings of Inability* (*n* = 5), *Shyness* (*n* = 5), *Financial Situation* (*n* = 5), *Family problems* (*n* = 5), *Guilty* (*n* = 4), *Anger* (*n* = 4), *Emotional Abuse* (*n* = 3), *Anxiety* (*n* = 3), *Feelings of Regret* (*n* = 3), *Feelings of Inferiority* (*n* = 3), *Intolerance* (*n* = 3), *Others' well fare* (*n* = 3), *Anguish* (*n* = 2), *Accidents* (*n* = 2), *Neglect* (*n* = 2), *Emotional Dependency* (*n* = 2), *Depression* (*n* = 2), *Physical/Emotional Pain* (*n* = 2), *Frustration* (*n* = 2), *City Moving* (*n* = 2), *Alcohol/Drug Abuse* (*n* = 2), *isolation and solitude* (*n* = 2), *selfishness* (*n* = 1), *bad habits* (*n* = 1), *bitterness* (*n* = 1), *negativity* (*n* = 1), *pride* (*n* = 1), *sloth* (*n* = 1), *resentment* (*n* = 1), *missing someone* (*n* = 1), *health status* (*n* = 1) and *suicide ideation* (*n* = 1).

For the moment post-COVID-19 (Fig. 2), 18 categories stood out: *Grief* (*n* = 5 citations), *Anger* (*n* = 3), *Anxiety* (*n* = 3), *Fear* (*n* = 3), *Sadness* (*n* = 3), *Trauma* (*n* = 2), *Relationships* (*n* = 2), *Physical/Emotional Pain* (*n* = 2), *Grudge* (*n* = 2), *COVID-19 pandemic* (*n* = 2), *Accidents* (*n* = 2), *Alcohol/drug abuse* (*n* = 1), *Anguish* (*n* = 1), *Depression* (*n* = 1), *Distrust* (*n* = 1), *Family Situation* (*n* = 1), *Feelings of inferiority* (*n* = 1), *Health status* (*n* = 1).

Regarding personal aspects the participants would like to change (Fig. 3), at the moment of Pre COVID-19 pandemic, 25 categories stood out: *shyness* (*n* = 10), *prioritising itself* (*n* = 6), *health status* (*n* = 6), *social interaction* (*n* = 5), *being more patient* (*n* = 4), *nothing* (*n* = 4), *improve self-esteem* (*n* = 4), *being more active* (*n* = 4), *the way of thinking* (*n* = 4), *temperament* (*n* = 3), *Physical appearance* (*n* = 3), *being more joyful* (*n* = 3), *being more comprehensive* (*n* = 2), *attain more knowledge* (*n* = 2), *develop listening* (*n* = 2), *be calmer* (*n* = 2), *improve work competences* (*n* = 2), *distrust* (*n* = 1)*, fear about losing siblings* (*n* = 1)*, fear the future and ageing* (*n* = 1)*, being more active in society* (*n* = 1)*, being more independent* (*n* = 1)*, being more persistent* (*n* = 1)*, being more resilient* (*n* = 1) and *anxiety* (*n* = 1). When looking at post-COVID-19 pandemic, eight categories stood out: *the way of thinking* (*n* = 3), *prioritizing itself* (*n* = 3), *health status* (*n* = 2), *change looks* (*n* = 2), *being more joyful* (*n* = 1), *temperament* (*n* = 1), *being more active* (*n* = 1) and *improve self-esteem* (*n* = 1).

**Fig. 4:**
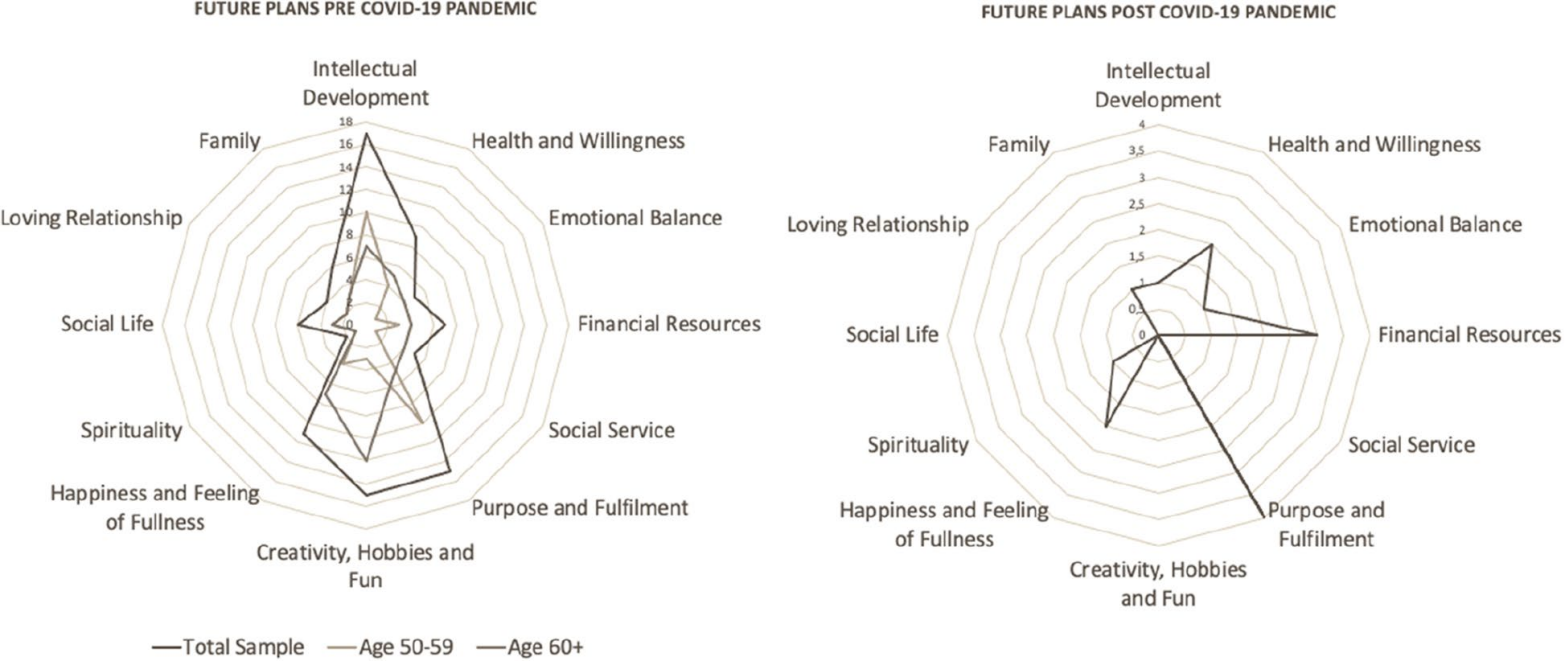
Wheel of Life’s representation of the resulting plans pre and post-COVID-19 pandemic from the answers given by the participants of the study. Fonte: Own authorship, Brazil, 2022

The wheel of life’s results is presented in Fig. 4, using the visual representation of a radar chart highlighting *Intellectual Development* (*n* = 17), *Purpose and Fulfilment* (*n* = 16) and *Creativity, Hobbies and Fun* (*n* = 15) at the moment Pre COVID-19 pandemic. Post COVID-19, *Purpose and Fulfilment* (*n* = 4) and *Financial Resources* (*n* = 3) were the most cited categories.

77% (PRE moment) and 97% (POST moment) of the participants' speeches about *feelings, experiences, and situations to be left behind* (Fig. 2) were directly linked to their personal plans (Fig. 4) and about *personal aspects, the participants would like to change,* 62% (PRE moment) and 90% (POST moment) were linked. This result demonstrates that, especially at the post-COVID-19 moment, the feelings, experiences, situations, and personal aspects they would like to change are linked to their plans.

70% of our post-COVID sample kept their previous speeches focused on their personal plans, some of them adding new information. 40% of that sample also related their plans to COVID-19 situations, and 80% changed their address about feelings and situations to leave behind.

The content from participants' personal plans answers is presented below in Table 2.

**Table 2:** Personal plans participants' answers

Categories	Participant’s Answers
Intellectual Development	*“My biggest wish is to graduate, wear the graduation robe and to attain a college degree[…]”*
Health and Willingness	*“[…] Change my way of life, eat better, doing more exercises.”; “Taking better care of health.”*
Emotional Balance	*“worry more about myself and less about others.”; “I want to be more coherent […]”; "Realising my internal transformation; accepting myself as I really am […]”*
Financial Resources	*“[…] planning to return to work as a career for the elderly.”; “regulate financial situation.”*
Social Service	*“[…] Even as a volunteer I want to work.”; “Helping my fellow man”*
Purpose and Fulfilment	*“[…] Opportunity to work in the area of seniors, if possible, improving myself in this area.”; “I want to work too. I don’t want to stand still.”; “I’m retired but I still want to work. I still have a future.”; “Become independent; to get professional”*
Creativity, Hobbies and Fun	*“[…] Having fun, dancing, traveling and a lot of recreation.”; “[…] keep travelling a lot”; “[…] Travel, more still as long as the legs hold out.”*
Happiness and Feeling of Fullness	*“To continue as I am, taking care of myself, with joy and happiness.”; “Enjoying the fruits of what has been achieved.”*
Spirituality	*“Peace.”; “Thanking God for every minute of my day”*
Social Life	*“To relate better with people; to enjoy life with more lightness and be able to celebrate more with people; to learn and pass a little of my experience to other people.”*
Loving Relationship	*“Get married and be happy”; “[…] getting a good mate for a good relationship.”*
Family	*“[…] enjoy my daughters”; ‘To be a very present grandfather at the family level.”*

## Discussion

Among the categories that had the most citations in the pre COVID-19 “personal plans” question (*Intellectual Development, Purpose and Fulfilment* and *Creativity, Hobbies and Fun*—Fig. 4), only *Purpose and Fulfilment* was kept in the post COVID-19 moment. After COVID, the participants' plans were mostly related to the Professional Sphere of the wheel.

It’s important to highlight that, within the four main aspects on the wheel, *Relationships* is the only one that was not present as a major guideline in the participants’ elaboration of personal plans, neither pre or post moment. Furthermore, our data about the plans doesn’t agree with results attained in other researches that tried to evaluate senior’s expectations for the future, in which the most present categories found were “*Uncertainty and Conformity*”, “*Pessimism*” and “*Longevity”* [19, 28]*.*

In Silva’s study [28], the future expectations related to *accomplishing goals* were limited to the concept of maintaining good body health, which was also mentioned in this study (Fig. 3, *focusing on health* and *being more active*; Fig. 4, item *Health and Willingness*), but in a lesser extent. Health was also mentioned in participants' plans at the post COVID-19 moment, not under a survivor perspective, but as a part of their lives that needed to be balanced.

Our research results, however, differs from Silva’s when our participants plans are related to their perception regarding the importance of education (Fig. 4, item *Intellectual Development*), held as a paramount element in the elaboration of personal plans and associated with professional growth (Fig. 4, item *Purpose and Fulfilment*). Even post-COVID-19, participants still wanted to archive professional goals.

It’s interesting to notice the shift, since our study shows that people of advanced age no longer focus only on body health as a goal. With a new perspective, reflected in the numerous possibilities of growing old, education seems like a major component in their lives, strengthening the idea that it is a relevant alternative to promote an active growing [29], either as a tool for professional goals (as in the answers *“Specialize myself*” and “*Completing my college*”) or as a search for knowledge in this stage of life (as in the answers “Studying more” and “Acquiring more knowledge”*).* By being students in an educational program focused on people of advanced age, it seems that our participants sample already invested in the achievement of this sphere of their personal plans, which is supported by other research that indicate older citizens’ inclusion in education [30].

Although Brazilian’s educational system emphasizes on preparing people to the job market, people of older age keep wishing to *attain more knowledge* (Fig. 3), even though it doesn’t necessarily mean more employability or income. The educational model that focus on preparing people to professional practice, although entrenched in Brazilian’s classrooms [29, 31], seems to be ineffective and incomplete for 50 + people's needs our results highlight, that it does not cover the educational wishes highlighted for lifelong learning opportunities nowadays. Nevertheless, our participants' sample seeks education as a fulfilment goal and also better financial conditions, especially during the pandemic (Fig. 4).

Working with 50 + people’s data brings several challenges, because of this population risk in bio-psychosocial aspects and, additionally, because of the heterogeneity of profiles on these people [32–34]. This heterogeneity, either it is biological, social or economic, shows, especially in Brazil, the high inequality of aging in the country, associated with the lack of public policies that take in account the several nuances of this citizens.

In contrast, we have the psychosocial data gathered on this research. The participants, although very different in biological and financial situations, walk through the same social aspects as sadness, solitude, family neglect, dependency, feeling of need to prioritize themselves, among others (Fig. 2 and 3) in their answers about things they want to leave behind and future plan guides (at pre and post COVID-19 moments). These answers are often highlighted in other researches with different populations [35, 36].

We could observe in this study consistent results with what other researchers found about the lack of family and the discontent with the family situation (Fig. 2) [37]. Answers like “*lack of family interaction with mother and father”*, *“Family rejection”,* “*neglect/feeling of loss*” “*empty nest*” reaffirm a sad reality where neglect is the most frequent form of abuse in Brazilian families [38].

Consequently, it’s not surprising the low number of references to family in the participants' plans pre COVID-19 (Fig. 4) and the fear of solitude in old age (Fig. 2 item *general fear; loneliness* and *isolation* within the *Others* category in feelings, experiences and situations to be left behind). Loneliness has also been identified as a worrying factor facing the pandemic, since it can be aggravated by social isolation and mandatory distancing of family members [11, 39, 40].

Despite their right to be supported by their siblings, secured in Brazil's Constitution, and the steadfast discussions to develop strategies to fight old peoples’ abuse, the number of elders neglected by their families increases [41]. The lack of effective public policies that reflect the particularities of old age contributes to this number since there is no support for the family to practice the needed care, taking into account that many family members don’t have the financial or psychological conditions to fulfil the role as a caretaker. COVID-19 family losses have exacerbated this situation and, according to Camarano, Pasinato e Machado [38]*,* one aggravating factor is that nowadays, women, traditionally viewed as the caretaker, are also participating in the labour market, which restricts possibilities of taking care of dependent family members.

This could also explain the results related to the item *Purpose and Fulfilment,* since the sample was comprised in its most part by women (76%), which suggests the need to have a closer look at the generation changes partaken by those women.

The rights conquered by the feminist struggle caused an impact in the perception of women about their social and economic role in society. While some years ago, despite the constant fight for rights, they were subordinates to patriarchal control [42], being deprived of work and practice of a major number of professions, today, women became economically independent and highly active in the labour market, contributing with a significant share of it.

In this scenario, 50 + women want now to do what they were once denied: to stop prioritizing other people (Fig. 2, item *focusing on others’ well fare*), to work (Fig. 4, item *Purpose and Fulfilment*; Fig. 4, item *Improve work capacity*), to achieve professional fulfilment (Fig. 2, item lack of professional achievements) and to establish relationships that are not loving related (Fig. 3, item *changing the way to relate with others*; Fig. 4, item *loving relationships*), understanding that they have power over their choices and authority to prioritize themselves (Fig. 3, item *Prioritizing itself*).

Answers related to relationships in Fig. 2 show dissatisfaction with matrimonial choices and are mostly related to traumas lived while they are married, which follows the results found on other studies that focus on this matter and corroborates to the increase of divorce number’s around the world [37, 43, 44]. Also, few references to *loving relationships* on participants’ plans (Fig. 3) refer to a situation where they do not seem to wish having a new loving relationship. Nevertheless, participants recognize the necessity of change for new emotional bonds to be made (Fig. 3. Item *change the way to Interact with others*), accepting that they also had a part in unsuccessful relations that they wish to leave behind (Fig. 2) and that there are still relations, especially related to family, that still need their attention. This was highlighted when we observed post-COVID-19 speeches, in which family rescue and valorisation were cited by the participants.

Therefore, *Development of listening skills* and the need to *be more comprehensive* (Fig. 3) appears highlighted on participants’ answers as vital changes to allow the growth of healthier relationships. Especially when facing COVID-19, people find themselves in a situation where they need to be isolated at home, some with the constant company of family members sharing rooms for weeks. Despite being challenging, the time that family members can enjoy during isolation can be an opportunity to improve and strengthen relationships.

Social life, despite being constantly reflected in other studies as a determinant factor to help avoid isolation and depression [45], did not stand out on the participants’ personal plans (Fig. 4). It’s important to emphasize that collaborators on the studied sample are already part of an educational program where social interaction happens daily, even in remote mode. As other studies showed, social interaction with online resources during COVID-19 was essential to reduce the impact of strict social isolation.

Nevertheless, it’s worth mentioning that shyness (Fig. 2; Fig. 3 item *being less shy*) was highlighted as an aspect to be overcome, showing the necessity of empowering these individuals through development of their potential to have an active voice and capacity of self-appreciation. The emphasis on self-esteem growth is reinforced by answers like *to change looks*, *to have a better self-esteem* (Fig. 3) and *Feelings of Inability* (Fig. 2), aspects that require a special attention, being a stage of life that brings relevant changes on appearance, physical and psychological abilities and, also, in society’s opportunity offers of full development of the human being.

Grief (Fig. 2) is often significant in any stage of life, considering it as a natural reaction to the loss of a loved one [46]. However, it acquires a much larger importance as long as you had lived, as the losses experienced in human’s lives become more frequent and represent the end of social, work and family affective relationships nurtured over the years [47] in a stage that approaches the finitude of the self-existence, where death becomes an explicit possibility. It is important to highlight that this is expanded while the world is experiencing a period of collective grief for the victims of COVID-19. Our post COVID-19 data showed a huge concern about it and the wish to leave grief experiences behind (Fig. 2).

Although other researchers found emotional balance essential to the maintenance of life quality [48] and improves cognitive functions, health care and symptoms of anxiety and depression [49], it was quoted few times in the personal plans’ answers in this study (Fig. 4). Meanwhile, participants mentioned in their answers the wish to overcome *traumas*, *fear*, emotional dependency, *abuse*, *anguish*, *depression*, *pain, frustration*, *anxiety, feelings* of *inferiority*, *intolerance, guilty* and *anger* (Fig. 2), in addition to seeking changes related to their *temperament*, to *being more patient,* to *being calmer* and *change the way of thinking* (Fig. 3).

Spirituality, (Fig. 4), often mentioned as an important aspect to be addressed in old age [50] did not have relevant room in participants’ answers.

Plans can diverge according to age level. In our study, our sample mixed ages between 50 and 81 years, but just a few differences were expressed. It was noted that the personal plans in younger older people in the sample were directed primarily to the professional area, while with the older group, the plans were directed to the area of creativity and hobbies, corroborating with other studies that highlight leisure activities as one of the priorities in old age [51].

31% (pre COVID-19) and 40% (post COVID-19) of the speeches about *personal aspects participants would like to change* (Fig. 3) were related to their personal plans, most of them interconnected by the theme of self-prioritization and professional field. When we compare feelings, experiences and situations participants would like to leave behind (Fig. 2), 23% (pre COVID-19) and 10% (post COVID-19) of the speeches were related to their personal plans, associated with themes like relationships and financial status. These results show the importance of understanding the changes participants want to make in themselves, once it seems to influence their decision about personal plans.

## Conclusion

In light of the foregoing, even though feelings are determinant to the paths of life of human beings since they “*translate the state of life in the language of the spirit”* [52], we observed that the feelings to be left behind by the collaborators did not seem to directly define the path in the elaboration of personal plans. Nevertheless, it influences their reflections about the aspects to change about themselves and promote resilience, in addition to providing a new look on their own lives, through the possibility’s lenses.

This research supports the findings on key aspects on aging profiles on Brazil and the world, such as the absence of the family; the necessity of reflection and rethinking of public policies – which are scarce and insufficient to meet the needs of this population – and the absence of high-quality education, a fundamental right and a tool in the maintenance of an active, healthy and dignified life.

When considering the impacts of the COVID-19 pandemic, it is relevant that more studies be carried out with other populations' ages/profiles to understand the perception of personal plans of people who have experienced the changes imposed by the pandemic, and how much it affects the construction of future’s personal planning.

This study has limitations related to the specific population included within the sample, as some of the participants were starting their path as part of an educational program for senior citizens and some studies indicate that people who search for education at an older age have a different motivation than general population [53–56]. Also, the sample was relatively homogeneous in terms of sex, while moderately different in terms of educational level. Future studies should explore other 50 + population groups (such as those living with their kids or other family members, those living in nursing homes/elderly care facilities, and those living on their own.). It is necessary to apply the same methodology to larger and more heterogeneous samples.

## Supplementary Information

**Additional file 1.** Supplementary file.

## Data Availability

The datasets used and/or analysed during the current study are available from the corresponding author on reasonable request, once the qualitative data can contain personal information of the participants.
