# Supplementary material for: COVID-19 and the elaboration of personal plans in + 50: a Brazilian experience
Source: BMC Public Health. 2023 Feb 1;23:221. doi: 10.1186/s12889-023-15006-1 (PMC9891749; doi:10.1186/s12889-023-15006-1)
Supplement: Supplementary file 1 — Additional file 1. Supplementary file. [file 12889_2023_15006_MOESM1_ESM.docx]

Supplementary file

Summary of the questions, categories and responses in Brazilian Portuguese.

1. Questionnaire Questions in English and Portuguese versions (the exact words).

| English | Portuguese |
| --- | --- |
| Question 1. Which feelings, experiences and situations would you like to leave behind? | Questão 1. Quais sentimentos, experiências e situações você gostaria de deixar para trás? |
| Question 2. What would you like to change about yourself? | Questão 2. O que você gostaria de mudar em você mesmo? |
| Question 3. What are your plans for the future? | Questão 3. Quais são os seus planos para o futuro? |

2. Portuguese version of the categories resulting from the sample responses on feelings, experiences and situations to be left behind, pre- and post-pandemic of COVID-19:

| English | Portuguese |
| --- | --- |
| Relationships (n=17 citations), Sadness (n=13), Family Absence (n=7), Grief (n=7), Trauma (n=6), Fear (n=6), Professional Frustration (n=5), Feelings of Inability (n=5), Shyness (n=5), Financial Situation (n=5), Family problems (n=5), Guilty (n=4), Anger (n=4), Emotional Abuse (n=3), Anxiety (n=3), Feelings of Regret (n=3), Feelings of Inferiority (n=3), Intolerance (n=3), Others' well fare (n=3), Anguish (n=2), Accidents (n=2), Neglect (n=2), Emotional Dependency (n=2), Depression (n=2), Physical/Emotional Pain (n=2), Frustration (n=2), City Moving (n=2), Alcohol/Drug Abuse (n=2), isolation and solitude (n=2), selfishness (n=1), bad habits (n=1), bitterness (n=1), negativity (n=1), pride (n=1), sloth (n=1), resentment (n=1), missing someone (n=1), health status (n=1) and suicide ideation (n=1). For the moment post COVID-19 (Figure 2), 18 categories stood out: Grief (n=5 citations), Anger (n=3), Anxiety (n=3), Fear (n=3), Sadness (n=3), Trauma (n=2), Relationships (n=2), Physical/Emotional Pain (n=2), Grudge (n=2), COVID-19 pandemic (n=2), Accidents (n=2), Alcohol/drug abuse (n=1), Anguish (n=1), Depression (n=1), Distrust (n=1), Family Situation (n=1), Feelings of inferiority (n=1), Health status (n=1). | Relacionamentos (n=17 citações), Tristeza (n=13), Ausência Familiar (n=7), Luto (n=7), Trauma (n=6), Medo (n=6), Frustração Profissional (n=5), Sentimentos de Incapacidade (n=5), Timidez (n=5), Situação Financeira (n=5), Problemas familiares (n=5), Culpa (n=4), Raiva (n=4), Abuso Emocional (n=3), Ansiedade (n=3), Arrependimento (n=3), Sentimentos de Inferioridade (n=3), Intolerância (n=3), Priorizar o bem dos outros (n= 3), Angústia (n=2), Acidentes (n=2), Negligência (n=2), Dependência Emocional (n=2), Depressão (n=2), Dor Física/Emocional (n=2), Frustração (n=2), Mudança de Cidade (n=2), Abuso de Álcool/Drogas (n=2), Isolamento e solidão (n=2), egoísmo (n=1), maus hábitos (n=1), amargura (n=1), negatividade (n=1), orgulho (n=1), preguiça (n=1), ressentimento (n=1), saudade de alguém (n=1), estado de saúde (n=1) e ideação suicida (n=1). No momento pós-COVID-19 (Figura 2), destacaram-se 18 categorias: Luto (n=5 citações), Raiva (n=3), Ansiedade (n=3), Medo (n=3), Tristeza (n=3), Trauma (n=2), Relacionamentos (n=2), Dor Física/Emocional (n=2), Rancor (n=2), Pandemia de COVID-19 (n=2), Acidentes (n=2), Abuso de álcool/drogas (n=1), Angústia (n=1), Depressão (n=1), Desconfiança (n=1), Situação Familiar (n=1), Sentimentos de inferioridade (n=1), Estado de saúde (n=1). |

3. Portuguese version of the personal aspects that participants would like to change, pre and post COVID-19 pandemic:

| English | Portuguese |
| --- | --- |
| Shyness (n=10), prioritising itself (n=6), health status (n=6), social interaction (n=5), being more patient (n=4), nothing (n=4), improve self-esteem (n=4), being more active (n=4), the way of thinking (n=4), temperament (n=3), Physical appearance (n=3), being more joyful (n=3), being more comprehensive (n=2), attain more knowledge (n=2), develop listening (n=2), be calmer (n=2), improve work competences (n=2), distrust (n=1), fear about losing siblings (n=1), fear the future and ageing (n=1), being more active in society (n=1), being more independent (n=1), being more persistent (n=1), being more resilient (n=1) and anxiety (n=1). When looking at post-COVID-19 pandemic, eight categories stood out: the way of thinking (n=3), prioritizing itself (n=3), health status (n=2), change looks (n=2), being more joyful (n=1), temperament (n=1), being more active (n=1) and improve self-esteem (n=1). | Timidez (n=10), priorizar-se (n=6), estado de saúde (n=6), interação social (n=5), ser mais paciente (n=4), nada (n=4), melhorar a autoestima (n=4), ser mais ativo (n= 4), o modo de pensar (n=4), temperamento (n=3), aparência física (n=3), ser mais alegre (n=3), ser mais compreensivo (n=2), alcançar mais conhecimento (n=2), desenvolver a escuta (n=2), ser mais calmo (n=2), melhorar as competências de trabalho (n=2), desconfiança (n=1), medo de perder parentes (n=1), medo do futuro e da velhice (n=1), ser mais ativo na sociedade (n=1), ser mais independente (n=1), ser mais persistente (n=1), ser mais resiliente (n=1) e ansiedade (n=1). No pós-pandemia de COVID-19, 8 categorias se destacaram: o modo de pensar (n=3), priorizar-se (n=3), estado de saúde (n=2), mudar de visual (n=2), ser mais alegre (n=1), temperamento (n=1), ser mais ativo (n=1) e melhorar a autoestima (n=1). |

4. Table 2. Personal plans of the participants' responses - Portuguese version.

| English | | Portuguese | |
| --- | --- | --- | --- |
| Categories | Participant’s Answers | Categorias | Respostas dos Participantes |
| Intellectual Development | “My biggest wish is to graduate, wear the graduation robe and to attain a college degree[...]” | Desenvolvimento Intelectual | "Meu maior desejo é me formar, vestir a beca de formatura e obter um diploma universitário [...]" |
| Health and Willingness | “[...] Change my way of life, eat better, doing more exercises.”; “Taking better care of health.” | Saúde e Disposição | "[...] Mudar meu modo de vida, comer melhor, fazendo mais exercícios."; " Cuidar melhor da saúde." |
| Emotional Balance | “worry more about myself and less about others.”; “I want to be more coherent […]”; "Realising my internal transformation; accepting myself as I really am […]” | Equilíbrio Emocional | "me preocupar mais comigo mesmo e menos com os outros."; "Quero ser mais coerente [...]"; "Realizar a minha transformação interna; me aceitar como eu realmente sou [...]" |
| Financial Resources | “[…] planning to return to work as a career for the elderly.”; “regulate financial situation.” | Recursos Financeiros | "[...] planejando voltar ao trabalho com uma carreira voltada para os idosos."; " regular a situação financeira". |
| Social Service | “[…] Even as a volunteer I want to work.”; “Helping my fellow man” | Serviço Social | "[...] Mesmo como voluntário quero trabalhar."; " Ajudando o meu próximo" |
| Purpose and Fulfilment | “[...] Opportunity to work in the area of seniors, if possible, improving myself in this area.”; “I want to work too. I don’t want to stand still.”; “I’m retired but I still want to work. I still have a future.”; “Become independent; to get professional”. | Realização e Propósito | "[...] Oportunidade de trabalhar na área dos seniores, se possível, me aperfeiçoando nesta área."; "Quero trabalhar também. Não quero ficar parado."; " Estou aposentado, mas ainda quero trabalhar. Eu ainda tenho um futuro."; "Me tornar independente; para me profissionalizar". |
| Creativity, Hobbies and Fun | “[...] Having fun, dancing, traveling and a lot of recreation.”; “[…] keep travelling a lot”; “[…] Travel, more still as long as the legs hold out.” | Criatividade, Hobbies e Diversão | "[...] Me divertindo, dançando, viajando e muita recreação."; "[...] continuar viajando muito"; "[...] Viajar, mais ainda, desde que as pernas se mantenham." |
| Happiness and Feeling of Fullness | “To continue as I am, taking care of myself, with joy and happiness.”; “Enjoying the fruits of what has been achieved.” | Felicidade e Plenitude | "Continuar como sou, cuidando de mim mesmo, com alegria e felicidade."; "Aproveitando os frutos do que foi alcançado." |
| Spirituality | “Peace.”; “Thanking God for every minute of my day” | Espiritualidade | "Paz"; "Agradecendo a Deus por cada minuto do meu dia" |
| Social Life | “To relate better with people; to enjoy life with more lightness and be able to celebrate more with people; to learn and pass a little of my experience to other people.” | Vida Social | "Me relacionar melhor com as pessoas; aproveitar a vida com mais leveza e poder celebrar mais com as pessoas; para aprender e passar um pouco da minha experiência para outras pessoas." |
| Loving Relationship | “Get married and be happy”; “[…] getting a good mate for a good relationship.” | Relacionamento amoroso | "Casar e ser feliz"; "[...] conseguir um bom companheiro para um bom relacionamento." |
| Family | “[…] enjoy my daughters”; ‘To be a very present grandfather at the family level.” | Família | "[...] aproveitar minhas filhas"; "Ser um avô muito presente a nível familiar." |
